# Intergenerational Resource Transfer Patterns between Parents and Children in South Korea and Depression

**DOI:** 10.3390/healthcare11081100

**Published:** 2023-04-12

**Authors:** Junpyo Kim, Yongho Chon, Myoung-il Kim

**Affiliations:** 1 School of Social Welfare, Gyeongsang National University, Jinju 52828, Republic of Korea; 2 Department of Social Welfare, Incheon National University, Incheon 22012, Republic of Korea; 3 Institute of Human Rights and Social Development, Gyeongsang National University, Jinju 52828, Republic of Korea

**Keywords:** intergenerational resources transfer, older parents, children, depression, latent class analysis

## Abstract

This study investigated the patterns of intergenerational resources transfer between parents and children in South Korea, and the influence on depression by its patterns. To maintain this, the seventh wave of Korean Longitudinal Study of Aging data were used. For data analysis, Latent Class Analysis (LCA) was used with five sub-factor variables: direct and indirect connections, receiving and providing financial support, and rearing grandchildren. For additional analysis, crosstab, logistic analysis, Analysis of Variance (ANOVA), and multiple regression were used. In the results, the optimal number of latent classes was four (parents offering, financial-centered, mutual offering, and emotional and financial-centered). In addition to the LCA results, there were some differences in predictors of pattern determination in each country. According to the results of ANOVA and multiple regression, parents offering and financial patterns led to more depression than the other two patterns. Based on the results, the importance of mutual communication and emotional connection was suggested for managing depression in South Korean older parents.

## 1. Introduction

According to the statistics from World Health Organization (WHO), about 7% of older people have depressive feelings and this accounts for 5.7% of health problems in the years lived with a disability [1]. From the survey of living conditions and welfare needs of Korean older persons in 2020, 33.1% of Korean older persons answered that they have depression, and this ratio goes higher as they get older [2]. Based on these statistics, depression in older persons is quite common but needs to be considered seriously because it is related to many elements of life including quality of life [3,4].

Among the suggestions on elderly depression, relationships between parents and children are important, and firmly maintaining these relationships can be helpful in these individuals’ later lives [5,6]. According to the convoys of social relations model, older people tend to narrow the range of social partners who give them emotional satisfaction in their relationships, so they put stress on micro relationships, such as those with family or friends [7]. In the same way, maintaining relationships with parents can also be helpful for children to prepare them to be independent in their young adult period [8,9]. Thus, relationships between parents and children can have mutual reciprocity.

Intergenerational resource transfer can be considered a way of cementing these relationships, and it can be explained using altruism theory. According to Kohli and Künemund [10], altruism theory in intergenerational transfer supposes parents to be altruistic breadwinners and children to be egoistic in households, and vice versa. An altruistic person can earn his or her well-being by raising another’s well-being. Applying this to family relations, an altruistic breadwinner who attempts to support their family can raise their well-being not only by consuming goods for him or herself but also by supporting goods for their family, so this action enhances the well-being of other people who are the target of resource transfer, as well as the family donor [10,11]. In intergenerational resource transfer between parents and children, some cultural differences can be found between Western and Eastern cultures [9]. Eastern cultures can be described as collectivistic and as influenced by Confucianism, so more intimate bonds between families are found, as well as the recognition of duties that satisfy the well-being needs of family members [12,13,14]. However, this sense of duty is growing faint due to the societal change toward segmentation and advancement, which can be seen in Western countries [15,16]. Additionally, the age of independence is quite different among countries; Korean young adults tend to stay with their families until their employment due to the highly competitive and unstable job market after graduation, and many Koreans wish to experience university education [17,18]. Thus, in this global society, this study can be used to understand Eastern culture-based older people in Western countries.

Based on existing studies, researchers have found several subcategories in intergenerational resource transfer between parents and children [19,20,21,22]. The first is economic resource transfers between parents and children, which can be considered the traditional resource transfer between them. In the United States, many cases of financial transfers go from parents to children who are experiencing hardship, but in other countries, such as those in East Asia, this is characterized as care duties for elderly parents and might demonstrate a different appearance [23,24]. Next, emotional transfers can be described as regular physical contact and non-physical contact, such as phone calls and text messages [25,26]. Physical contact between parents and children is the most powerful emotional connection between them, and non-physical devices can provide technological connections despite the physical distance that means individuals cannot visit each other easily. Today, there are emerging needs of transferring human resources, such as grandchild care and housework. Unlike financial transference, housekeeping and caring tend to go from adult children to parents, but there are also cultural differences between Eastern and Western cultures [15,27].

In the relationship between intergenerational resource transfer and depression, many existing studies suggest that an equal relationship between parents and children leads to lower depression. On the contrary, a lopsided relationship between either parents or children can be stressful and may evoke a depressive mood [28,29,30]. However, these studies investigated the relationship as a variable; many of them used sub-categories of resource transfer such as financial exchange or subjective questions of transfer.

There are several existing studies about intergenerational resource transfer among families, but many of them focus on each transfer category rather than aspects of the entire transfer. To explore this, it is important to investigate intergenerational resource transfer as a whole, from person to person, not by each variable. Additionally, it is important to figure out the impact of each sub-type of intergenerational resource transfer on depression. This study explores both the patterns of intergenerational resource transfer in South Korea using latent class analysis (LCA) and its effects on depression by patterns.

## 2. Materials and Methods

### 2.1. Data

To investigate the intergenerational resource transfer patterns of parents and children in South Korea, the 7th wave of the Korean Longitudinal Study of Aging (KLoSA), which was conducted in 2018, was used. The KLoSA includes national panel data and was established in 2006. It surveyed 10,524 older adults using computer-assisted personal interviews, where each enumerator visited each home of the survey panels and filled out answers. To decrease the bias from the exact amount or degree of resource transfer, the answers were collected from parents only. Additionally, researchers viewed the time when a child becomes an adult and intergenerational resource transfer between parents and children begins to be 55 years old of parents because the average independence age of children is about 23 (male: 23.9, female: 23.5) [31]. For this reason, parents who were selected for data analysis were people over age 55 in the data set, and 5099 South Korean older adults were selected.

### 2.2. Measurement

#### 2.2.1. Variables for Resource Transfer Patterns

To examine the pattern of resource transfer between parents and children, five sub-variables were selected from the KLoSA data. The variables consisted of direct and indirect connections, receiving and providing financial support, and rearing grandchildren. First, direct connection was measured using the frequency of physical meeting between parents and children, and indirect connection was measured using the frequency of phone, text, and e-mail contact between them. Both of these variables were dichotomized based on their mean scores. Next, transferring financial support was measured dichotomously regarding whether parents received financial support from, or provided it to, children. Third, grandchildren rearing was measured using a question about the experience of grandchild rearing.

#### 2.2.2. Depression

To measure depression in Korean older parents, CES-D-10 (The Center for Epidemiologic Studies—Depression Scale) was used. The scale was originally invented by Kohout, Berkman, Evans, and Cornoni-Huntley [32], and translated into Korean by Chon, Choi, and Yang [33]. CES-D-10 consists of 10 questions with a 4-point Likert scale (0 = None of the time, 3 = All of the time), and if the score goes higher, it shows a more depressive mood. The cut-off score of this scale is 10, and the reliability (Cronbach’s Alpha) of the scale is 0.765.

#### 2.2.3. Socioeconomic Variables

To measure the socioeconomic characteristics of each pattern and influencing factor of the decision of pattern, socioeconomic variables such as gender, age, education, job, and religion were measured. Age was answered with the respondent’s year of birth and was recoded with their age based on 2018. The education of respondents was measured as elementary school or less, middle school, high school, and college or more. Job status and religion were answered and recoded with dichotomous variables.

### 2.3. Research Method

The analytical methods for this study used descriptive and crosstab methods for the characteristics of each variable and class. LCA was used to predict the latent class of resource transfer, multinomial regression was used to examine the class affiliation, and ANOVA and multiple regression analysis were used for the effects on depression by patterns. LCA is an analytical method that can explore latent classes of the variable that are mutually exclusive based on dichotomized data. LCA uses a person-based approach in which the unit of analysis is a case, not a variable, so cases that have similar characteristics are classified into the same class. In the decision-making process of the number of latent classes, LCA uses statistical indicators to predict the latent classes in a more objective and reliable way. For the indicators in this study, the Akaike Information Criteria (AIC), Bayesian Information Criteria (BIC), and sample-size adjusted BIC (SSABIC) were used, and the lower their values, the better the model. Additionally, entropy of the class—the average value that shows how correctly classified each case is—is correctly classified as closer to 1. To examine the statistical significance of the model, the Lo–Mendell–Rubin (LMR) test was used; if a *p*-value of the LMR test is not significant, the model cannot be used. Notwithstanding these indicators, the model decision should consider the usefulness, conciseness, and application possibility and distribution of each class, so it is a comprehensive process [34]. After LCA, the study investigated the sociodemographic factors on each pattern with crosstab and the determination factors on each pattern with multinomial logistic regression, so the practical social work implications based on sociodemographic factors in each pattern can be suggested. Next, ANOVA was performed to compare the CES-D scores on each pattern. Compared to the generalized linear model (GLM), ANOVA can focus directly on CES-D score comparison among patterns. In the analysis, the researchers used SPSS 24.0 for descriptive, crosstab, ANOVA, and regression analyses, and Mplus 8.0 was used for LCA.

## 3. Results

### 3.1. Latent Class Analysis of Resource Transference among South Korean Parents

The outcomes of the LCA are presented in Table 1. To determine the optimal latent class, the researchers put classes 2–5 into the model of resource transference. In the KLoSA (South Korea), the BIC value was consistently lower as the class number increased and was increased in class 5; this means that class 4 could be the optimal latent class. In addition to this, class 4 showed the highest entropy (0.955), so we decided that class 4 was the optimal latent class for the resource transference of Korean parents. In the distribution of each subclass, several classes showed low frequency, but based on Jung and Wickramara (2008), the distribution satisfied the minimal standard, and the subclasses showed unique characteristics, so the resource transference patterns of South Korean older parents were selected as a 4-class model.

### 3.2. Characteristics of Resource Transference Patterns

The characteristics of intergenerational resource transference of Korean older parents are shown in Table 2. In the case of South Koreans, the 4-class model was selected, and each subcategory was named based on its characteristics: offering by parents, financial-centered, mutual offering, and emotional and financial-centered. Resources in the offering by parent’s pattern moved from parents to children, and parents in the financial-centered pattern only received financial support from children. The mutual offering pattern showed high physical meeting or transfer with each other, except for the emotional link. Finally, the emotional and financial-centered pattern showed a high preference only for the emotional link and financial support from children.

### 3.3. Socioeconomic Characteristics of Each Pattern

The socioeconomic characteristics of the South Korean older parents in each pattern are shown in Table 3. There are several characteristics that can be divided among the patterns: gender, age, education, job status, religion, subjective health, and life satisfaction. Regarding the socioeconomic characteristics of the ‘offering by parents’ pattern, members of the pattern showed a higher percentage of being male, having higher education, and working. In the financial-centered pattern, there were several people aged 75+ who could not perform any financial activities. The mutual offering pattern showed a higher percentage of religious activity and life satisfaction, and the last group had many people currently centered on financial activities.

### 3.4. Predictors of Pattern Determination 

To investigate the factors that determine the difference between the four potential groups analyzed by the type of resource movement between parents and children, multinomial logistic regression was performed with the most popular pattern (emotional and financial-centered). The analysis results are presented in Table 4 (Chi-square: 281.283).

In the comparison between the emotional and financial-centered pattern and the offering by parent pattern in South Korea, gender, age, and education were the determinant factors that significantly affected the transference of patterns; women, older, and less educated members were more likely to fall into the emotional and financial-centered pattern. Next, age and job status were determinant factors between the emotional and financial-centered pattern and the financial-centered pattern; being younger and having no current job led to the emotional and financial-centered pattern. In the comparison between the emotional and financial-centered pattern and the mutual offering pattern, every factor except for age significantly determined transference; women, more highly educated, having no job, good health, and higher life satisfaction determined the mutual offering pattern.

### 3.5. Mean Comparison on CES-D 

To examine the relationship between resource transfer patterns and depression, an ANOVA was performed, and its results are shown in Table 5. For the depression score, the financial-centered pattern (7.78) showed the highest score among the four patterns, and the offering by parents pattern (7.71) followed. In the ANOVA results, it was determined that the model was significant (F = 5.565, *p* < 0.001). For depression score comparison, a post hoc test (Scheffe) was performed. Among the patterns, the financial-centered pattern (7.78) showed a significantly higher depression score than the mutual offering (6.52) and emotional and financial-centered patterns (6.74). However, among other patterns, there was no significance.

### 3.6. Multiple Regression with Patterns and Depression

Table 6 shows the results of multiple regression on depression by patterns. As a reference variable, the offering by parents pattern was selected and the rest of the patterns were recoded into dummy variables. Gender, age, education, job status, religion, and physical health were selected as control variables. In the results, every control variable in the model turned out to be significant (R^2^ = 0.114, F-value = 73.962, *p* < 0.001). Among the patterns, every dummied variable showed significance, so the offering by parents pattern members demonstrated higher depressive feelings than any other patterns.

## 4. Conclusions

This study aimed to investigate the intergenerational resource transference patterns between parents and children in South Korea by classifying the factors that influence their transfer and their socioeconomic characteristics and depression. To achieve this, the researchers examined KLoSA data using LCA, crosstab analysis, ANOVA, and regression analysis.

From the results of the LCA, we found the four patterns of intergenerational resource transference: (a) offering by parents, (b) financial-centered, (c) mutual offering, and (d) emotional and financial-centered. According to the results, intergenerational resource transference patterns in South Korea showed vertical differences centering on the mutual offering pattern, from much resource transference to focusing on a certain transference. In addition to this, each pattern had several determinants. South Korean parents showed more frequent and varied resource transference with their children, but this does not mean that South Koreans are more intimate than parents in other countries; this can be explained by the characteristics of preparation for elderly life in Korea. According to Nam, Kim, Shin, and Yim [35], later-life preparation in Koreans is not sufficient due to the immaturity of the pension system and the sudden change in elderly care culture such as the sense of duty. The Korean pension system was introduced in 1988 and has since passed its introduction, but its income replacement rate is still around 40% [36]. This means that older people in Korea must continuously participate in financial activities or be dependent on others, especially their children. Conversely, there are still needs for elderly and baby care placed on both older parents and their children, despite the increase in the demand for public care [35,37]. Compared to the United States, the age of independence in Korean children is much older, and many Korean older parents play a role in grandchild rearing.

These results are quite different from Western countries, and this difference might be due to the demographic differences in elderly age groups in each country, especially the Baby Boomer generation. The defined time of the Baby Boomer generation is slightly different between the US (1946–1963) and South Korea (1953–1963) due to the Korean War. Baby Boomers in Korea are entering the elderly period just now, but many Baby Boomers in the US are already in this period. There is about a 10-year gap between the two countries [35,38]. According to existing studies from the 1990s and early 2000s from the United States, the volume of intergenerational resource transference between parents and children was quite high; mutual resource transference and transference from children to parents happened frequently [14,20,36]. Based on this, the researchers carefully suggest that intergenerational resource transference in the US might be the future of South Korea. Thus, in South Korea, it might be useful to pursue this aspect of the United States’ past. Following the past in the US can predict what will happen in the relationship between parents and children in South Korea. Past experiences in some countries can be the solution to other countries’ upcoming troubles.

Next, from the results of ANOVA and multiple regression, patterns of resource transfer affected the depression of older parents. In detail, the financial-centered pattern showed a higher depressive mood than other patterns, and the offering by parents’ pattern influenced more negative effects than other patterns. This shows the importance of relationships and communication between parents and children. Some existing studies point out financial support as a common method of resource transfer, but this study result indicates that financial support alone is not enough to maintain sound mental health. With a harmonious combination of financial support, emotional connection, and mutual communication, an older parent can lower their depression. As an intervention for the elderly and family matters, strengthening relationships between children and parents in various ways can be a solution for their mental health.

According to these results, there are some implications that can be utilized both in South Koreans and Koreans in Western countries. As they provide an understanding of the difference in the patterns of resource transference between parents and children, the results can be used in the case of Koreans who live in the United States. Pointing out that Korean parents and children show more resource transference than other people, social issues that Koreans in the United States might have can be solved by enhancing the relationships with their children or parents. Additionally, the results can emphasize the cultural differences of those living in the United States. Even though the United States has flourished based on its cultural uniqueness, such as being a melting pot, it is difficult to dismiss one’s own cultural background. This result can be a base reference to understanding the cultural differences between Western and Eastern cultures and has implications for migrants from Eastern or Confucianism backgrounds.

There are a number of limitations in this study. First, KLoSA provides panel data that are limited to respondents over 55, so it was hard to measure the intergeneration resource transfer of both parents and children. Additionally, the number of sub-categories of intergenerational resource transfer was limited. Next, this study was conducted using cross-sectional data, making it difficult to confirm longitudinal changes due to resource exchange. For the next study, it is necessary to conduct a longitudinal study on how the type of support exchange with children changes during the aging process of the elderly.

## Figures and Tables

**Table 1:** Latent Class Analysis Results (n = 5099).

	Class 2	Class 3	Class 4	Class 5	Distribution (%)	
AIC	18,356.111	18,185.782	18,138.857	18,134.321	1	117 (2.3)
BIC	18,428.016	18,296.908	18,289.203	18,323.888	2	278 (5.5)
Adj. BIC	18,393.062	18,242.887	18,216.117	18,231.736	3	2061 (40.4)
Entropy	0.923	0.933	0.955	0.891	4	2643 (51.8)
LMR (*p*-value)	4226.715 ***	178.838 ***	57.797 ***	16.219 **		

*** *p* < 0.001, ** *p* < 0.01.

**Table 2:** Components of each class and naming.

		Latent Class			
		Offering by Parents	Financial-Centered	Mutual-Offering	Emotional and Financial Centered
Direct connection	Yes	0.000	0.029	0.067	0.019
	No	1.000	0.971	0.933	0.981
Indirect connection	Yes	0.196	0.000	0.629	1.000
	No	0.804	1.000	0.371	0.000
Receive fin. support	Yes	0.000	0.857	0.988	0.996
	No	1.000	0.143	0.012	0.004
Provide fin. Support	Yes	0.847	0.000	0.992	0.091
	No	0.153	1.000	0.008	0.909
Rearing gr. children	Yes	1.000	0.086	0.973	0.085
	No	0.000	0.914	0.027	0.915

**Table 3:** Sociodemographic Factors in Each Pattern.

		Patt. 1 (n = 117)	Patt. 2 (n = 278)	Patt. 3 (n = 2061)	Patt. 4 (n = 2643)	X2 (*p*-Value)
Gender	Male	69(59.0)	101(36.3)	721(35.0)	1108(41.9)	43.749 ***
	Female	48(41.0)	177(63.7)	1340(65.0)	1535(58.1)	
Age	Less than 65	51(43.6)	30(10.8)	429(20.8)	568(21.5)	94.067 ***
	65–74	29(24.8)	66(23.7)	766(37.2)	934(35.3)	
	75 or more	37(31.6)	185(65.5)	866(42.0)	1141(43.2)	
Education	Elementary or less	28(23.9)	173(62.2)	948(46.0)	1328(50.2)	80.890 ***
	Middle school	19(16.2)	48(17.3)	378(18.3)	469(17.7)	
	High school	48(41.0)	50(18.0)	557(27.0)	657(24.9)	
	College or more	22(18.8)	7(2.5)	178(8.6)	189(7.2)	
Job Status	Yes	56(47.9)	38(13.7)	524(25.4)	896(33.9)	93.102 ***
	No	61(52.1)	240(86.3)	1537(74.6)	1747(66.1)	
Religion	Yes	71(60.7)	176(63.3)	1159(56.2)	1724(65.2)	39.996 ***
	No	46(39.3)	102(36.7)	902(43.8)	919(34.8)	
Subjective Health		3.62	3.99	3.87	3.84	5.743 ** 1 < 2/3 2 > 1/4
Life satisfaction (%)		62.81	57.04	61.21	59.90	5.023 *** 2 < 4 < 1/3

*** *p* < 0.001, ** *p* < 0.01.

**Table 4:** Multinomial Logistic Regressions.

South Korea	1 vs. 4		2 vs. 4		3 vs. 4	
	B (s.e.)	OR	B (s.e.)	OR	B (s.e.)	OR
Intercept	−0.986(1.309)	-	−4.089(0.843)	-	−0.649(0.400)	-
Gender	0.473(0.218) *	1.605	−0.016(0.144)	0.984	−0.271(0.068) ***	0.762
Age	−0.043(0.014) *	0.958	0.036(0.008) ***	1.037	−0.005(0.004)	0.995
Education	0.418(0.106) ***	1.519	−0.146(0.080)	0.864	0.123(0.034) ***	1.131
Job Status	0.091(0.219)	1.096	−0.863(195) ***	0.422	−0.447(0.074) ***	0.640
Religion	−0.183(0.198)	0.833	−0.119(0.134)	0.888	−0.304(0.062) ***	0.738
Subjective Health	0.021(0.132)	1.022	−0.074(0.087)	0.928	0.111(0.041) **	1.117
Life Satisfaction	−0.004(0.008)	0.996	−0.001(0.005)	0.999	0.009(0.002) ***	1.009

*** *p* < 0.001, ** *p* < 0.01, * *p* < 0.05.

**Table 5:** Mean Comparison of CES-D Scores by Patterns.

Pattern Name	CES-D Scores (Mean = 6.73)	CES-D Cut-Off (Cut-Off Ratio = 28.4%)	F-Value (Post-Hoc: Scheffe)
Pattern 1 (Offering by parents)	7.71	42 (35.9)	5.565 ** (2 > 3, 4)
Pattern 2 (Financial-centered)	7.78	86 (30.9)	
Pattern 3 (Mutual offering)	6.52	568 (27.6)	
Pattern 4 (Emotion and financial Centered)	6.74	753 (28.5)	

** *p* < 0.01.

**Table 6:** Multiple Regression Analysis on Depression by Patterns ^1^.

Variables	B (s.e.)	ß	t (*p*-Value)
Gender	0.057(0.022)	0.037	2.546 *
Age	0.034(0.005)	0.103	6.352 ***
Education	−0.134(0.045)	−0.047	−2.991 **
Job-status	0.184(0.024)	0.114	7.614 ***
Religion	−0.505(0.081)	−0.083	−6.207 ***
Physical health	0.630(0.050)	0.183	12.568 ***
Patt. 2	−0.665(0.311)	−0.051	−2.139 *
Patt. 3	−0.913(0.266)	−0.151	−3.426 **
Patt. 4	−0.768(0.265)	−0.130	−2.903 **

Adjusted R^2^ = 0.114, F-value = 73.962 ***, ^1^ Reference variable: Offering by parents. *** *p* < 0.001, ** *p* < 0.01, * *p* < 0.05.

## Data Availability

KLoSA at https://survey.keis.or.kr/eng/index.jsp, accessed on 1 August 2022.
